# Isoliquiritigenin attenuates acute renal injury through suppressing oxidative stress, fibrosis and JAK2/STAT3 pathway in streptozotocin-induced diabetic rats

**DOI:** 10.1080/21655979.2021.2006978

**Published:** 2021-11-30

**Authors:** Leiming Sun, Zheng Yang, Jiaying Zhang, Jie Wang

**Affiliations:** Department of Critical Care Medicine, Hangzhou Red Cross Hospital, Hangzhou, Zhejiang Province, PR China

**Keywords:** Isoliquiritigenin, diabetic nephropathy, oxidative damage, fibrosis, TGF-β/Smad signaling pathway

## Abstract

The aim of the current study was to evaluate the protective effects and mechanisms of isoliquiritigenin (ISO) on acute renal injury. CCK-8 assays were applied to assess the effects of ISO at different doses (20, 40, and 80 μg/mL) on oxidative damage in human renal HK-2 cells incubated with high glucose. After the diabetic nephropathy (DN) rat model was established, the model animals were randomly assigned to saline-treated control, three model groups received the 10, 20 and 40 mg/kg ISO, respectively, using the healthy Sprague-Dawley (SD) rats as normal control. The blood biochemical indexes, renal functions, oxidative stress, morphological changes, fibrosis- and JAK2/STAT3-related factors in DN model rats were all assessed. The cellular viability of the renal HK-2 cells with oxidative damages were all markedly ameliorated via the incubation of ISO between 10 and 80 μg/mL compared with negative control. In addition, the significantly down-regulated ROS content and up-regulated expression levels of GSH, SOD2, and GPX1 were all observed in ISO-treated groups. Long-term administration of ISO at different doses in DN rats effectively improved general diabetic characteristics and renal morphology. Furthermore, long-term administration of ISO could ameliorate excessive oxidation stress, down-regulate the expression levels of renal fibrosis- and inflammation-related factors, as well as inhibit the JAK2/STAT3 signaling pathway. In conclusion, ISO at all three dosages could efficiently improve the renal injury induced by STZ via ameliorating renal fibrosis, oxidative stress, and inhibiting JAK2/STAT3 signaling pathways in the DN rats.

## 1. Introduction

Diabetic nephropathy (DN) is the most serious and harmful form of chronic renal microangiopathy among diabetic complications, with an incidence of ~ 30% in patients with diabetes [1–3]. Recently, DN has emerged as the first cause of end-stage renal disease and the leading cause of death in diabetic patients [1,4]. Therapeutical strategies for treating diabetes are continuously improving, while there is little effect on many chronic diabetic complications, such as cardiovascular and kidney diseases, and making DN the main cause of death and disability in patients with diabetes [5–8]. Therefore, the number of patients with DN caused by diabetes and end-stage renal failure caused by DN are both increasing year by year [9]. DN is clinically defined as a process in which excessive deposition of glomerular cells and ECM in the glomeruli and tubules leads to microalbuminuria to proteinuria [10]. Thickened glomerular and glomerular capillary basement membrane and increased matrix will ultimately lead to glomerular and tubulointerstitial fibrosis, and also ultimately lead to gradual and irreversible loss of renal function [3,11]. Moreover, renal fibrosis is not only a feature of disease progression, but also exacerbates the decline in renal function [12,13]. Despite numerous experimental reports on the study of DN, currently available strategies can only improve or delay the progression of chronic kidney disease but cannot reverse fibrosis [12,14]. The pathogenesis of DN has not yet been fully elucidated and most scholars demonstrated that the occurrence and development of DN is the result of a combination of multiple factors, such as increased oxidative products, glucose, and lipid metabolism disorders, under a long-term hyperglycemic environment [15–18].

Isoliquiritigenin (ISO) is a kind of flavonoid, which was extracted and isolated from the Chinese medicinal herb *Glycyrrhiza uralensis*, and its molecular structure belongs to hydroxychalcone compounds [19]. ISO exists in a variety of medicinal plants, especially in licorice, with multiple biological activities, including anti-tumor, anti-diabetic complications, and so on. Therefore ISO has become one of the research hotspots in the Chinese medicine community [20]. Park et al. used a rat model of acute liver injury induced by CCl4, and found that ISO held protective effects on liver injury in rats, and the mechanism was related to scavenging free radicals and anti-lipid peroxidation in liver tissue [21]. As a monomeric component of licorice flavonoids, ISO inhibits tumor necrosis factor-α (TNF-α) induced reactive oxygen species (ROS) generation in epithelial cells, prevents the TNF-α-induced accumulation of vascular cell adhesion molecule (VCAM-1) and E-selectin, attenuates excessive inflammatory responses [19,22]. ISO also holds antiplatelet aggregation effects which is similar to aspirin, inhibits cyclooxygenase, and reduces thromboxane A2 [23–25].

However, whether ISO could be applied in the treatment of DN has not been fully demonstrated. In this study, we hypothesized that ISO may trigger beneficial effects on DN in rodent animals with diabetic kidney injury. In support of this hypothesis, the protective effects of ISO (20, 40, and 80 μg/mL) on cell viability of high glucose induced HK-2 cells and the chronic efficacies of ISO (10, 20 and 40 mg/kg) on diabetic kidney injury in DN rats were both evaluated. In addition, the potent mechanisms were also investigated.

## 2. Material and methods

### 2.1 Materials and animals

The ISO (purity more than 98%) and CCK-8 kit were both brought from Beijing Chengke Biotech Co., LTD (Beijing, China). The commercialized monoclonal antibodies targeting the transforming growth factor β1 (TGF-β1) (Cas: bsm-33,345 M, liquid, 44 kDa), Collagen-1 (Cas: bs-0578 R, liquid, 130 kDa), fibronectin (Cas: bs-0666 R, liquid, 259 kDa), interleukin-6 (IL-6) (Cas: bs-4539 R, liquid, 23 kDa), intercellular adhesion molecule 1 (ICAM-1) (Cas: bs-4615 R, liquid, 56 kDa), JAK2 (Cas: bs-23,003 R, liquid, 131 kDa), phosphorylated-JAK2 (p-JAK2) (Cas: bs-3206 R, liquid, 131 kDa), STAT3 (Cas: bs-55,208 R, liquid, 88 kDa), phosphorylated-STAT3 (p-STAT3) (Cas: bs-1658 R, liquid, 85 kDa), B-cell lymphoma-2 (BCL2) (Cas: bs-34,012 R, liquid, 26 kDa), and BCL2-Associated X (BAX) (Cas: bsm-33,283 M, liquid, 21 kDa) were all obtained from the Beijing Bioss Biotechnology Company (Shanghai, China). Glutathi one (GSH) (Cas: 7512–100-K), ROS detection kits (Cas: 200–664-3) were obtained from R&D Com pany (USA). The blood urea nitrogen (BUN) (Cas: EIABUN), creatinine (Crea) (Cas: EIACUN), uric acid (UA) (Cas: MAK077), superoxide dismutase (SOD) (Cas: EIASODC), and malondialdehyde (MDA) (Cas: MAK085) kits were brought from Invitrogen Biotechnology Co., LTD (Utah, USA). β2-microglobu lin (β2-MG) (Cas: ab223590) kits were brought from Abcam (Cambridge, UK). BCA detection kit (Cas: P0012S) was acquired from Beyotime Biotech Co., LTD (Beijing, China). The human renal HK-2 cells (ATCC CRL-2190) were brought from American ATCC Cell Bank.

Forty female Sprague-Dawley (SD) rats, 5–7 weeks old, 160 ± 15 g, as well as SPF-grade high fat and normal feed were brought from Shanghai Sla ck Laboratory Animal Company (Shanghai, China). Three healthy rats per cage were kept under the temperature of 25 ± 2°C and relative humidity of 65%-75%. All the animals were exposed to the acclimatization period for 7 days. Animal experiments were applied according to standard procedures compiled by animal experiment center of SLbio Company (Shanghai, China) with approval No. CDI-202234-021.

### 2.2 Cell-based assays

HK-2 cells were cultured in DMEM medium and placed in the incubator (37°C, 5% CO_2_). When the cells grew well and fused to about 85%, the cells were digested and seeded in 6-well plates, and cells were given serum-free DMEM medium until they reached about 80%. Then, HK-2 cells were continuously cultured in DMEM medium (30 mmol/L glucose) at a density of 10^5^ cells/well to simulate the *in vivo* environment with high glucose, and finally establish a suitable DN cell model for *in vitro* evaluation. We further incubated ISO and cells at final concentrations of 2.5, 5, 10, 20, 40, 80, and 160 μg/mL. After 36 h co-treatment, 50 μL CCK-8 solutions were added for 4 h incubation. Absorbance of different groups at the wavelength of 450 nm were read via SpectraMax® iD5 (Molecular Devices, USA).

### 2.3 The measurement of supernatant content of ROS and GSH

The culture of high-glucose incubated HK-2 cells was performed as above described. After 24 h co-treatment with ISO at final concentrations of 20, 40, or 80 μg/mL, the cell culture supernatants were added with DCFH-DA at a final concentration of 10 mM. Further incubation at 37°C for half an hour, the HK-2 cells were washed for three times and then separated to detect fluorescence intensity via the flow cytometer (BD, USA). The GSH and SOD content in the HK-2 cell was measured according to instruction of kits.

### 2.4 Establishment of DN model rats and experimental design

The high-fat diet combined with intraperitoneal treatment of STZ (60 mg/kg) was applied to establish the DN rat model until both fasting BGL was stably >11.1 mmol/L and the 24 h urine proteins (UP/24 h) were over 30 mg. The model animals were randomly assigned to saline-treated control, three model groups received the 10, 20, and 40 mg/kg ISO, respectively, using the healthy SD rats as normal control. The healthy SD rats in the normal control and DN model groups received the normal and high fat feeding, respectively. The long-term treatments of ISO in DN model rats were conducted daily at the doses of 10, 20, and 40 mg/kg for 8 weeks. The mental status, activity, fur color, water intake, and urination and defecation of the animals were observed every day. Body weights were measured and recorded daily prior to dosing. At week 0 and 9, the diabetic rats were fasting for 4 hours, and then the serum samples were obtained to measure fasting blood glucose. At week 0 and 9, the rats were housed for 12 h of adaptive feeding, and 24 hours of urine was collected. Twenty-four hours urine volume and urine protein were measured, respectively. At week 9, rats were anesthetized by sodium pentobarbital and the blood samples were collected from abdominal aorta. Serum was separated by centrifugation at 3500 × g for 15 min to detect serum levels of total cholesterol (TC), triglycerides (TG), Blood Urea Nitrogen (BUN), high density lipoprotein (HDL), low density lipoprotein (LDL), and other biochemical indicators according to instruction of each kit or automatic blood biochemical analyzer (Beckman, Germany). Finally, we promptly separated the renal tissue of rats to analyze the kidney/body ratio, then divided into two parts. One part was immediately frozen in dry ice and stored at −80°C for further Western blotting analysis. The other part stored in the 4% paraformaldehyde solution for 24 h. The pathological morphology of kidney tissues from DN model rats were performed by using H&E staining methods. Sections were observed and imaged by microscope (model BX-53, Olympus Optical, Tokyo, Japan). The expression levels of fibrosis- and JAK2/STAT3 signaling pathways-related factors were measured by using the Western blotting method.

### 2.5 Western blotting detection

The total protein in the HK-2 cells and renal tissues were extracted and quantified via BCA method according the instruction. Quantified protein samples were further separated via the SDS-PAGE (15%), and transferred to PVDF membrane at the condition of 110 V, 1 h, and then incubated with 5% BSA as blocking buffer for 12 h at 4°C, and further incubated with different antibodies at the dilution ratio of 1:5000. The HRP-conjugated second antibody was diluted at the ratio of 1:5000 and then incubated with the PVDF membrane after being washed by using the PBST containing the 0.05% Tween-20. The follow-up coloration of PVDF membranes was conducted according to standard methods and then photo was taken by Tanon-5200 (Tanon, China). The bands of gel image were further analyzed via Image J.

### 2.6 Statistical analysis

Initially, all data were analyzed for normality (Shapiro-Wilk test) and homogeneity (Bartlett test). All the results were analyzed as Mean ± SD via the software of Graphpad Prism 8.4 using the method of one-way ANOVA. Tukey's test was used in post hoc analysis for ANOVA. *p < *0.05 was considered statistically significant.

## 3. Results

In this study, we hypothesized that ISO may trigger beneficial effects on DN in rodent animals with diabetic kidney injury. Firstly, we demonstrated that pretreatment of ISO could improve the cell viability of high-glucose induced HK-2 cell via the amelioration of oxidative stress. Furthermore, a chronic study in DN model rats revealed that long-term treatment of ISO could improve the diabetic characteristics and renal functions in acute DN rats through ameliorating renal fibrosis, oxidative stress, and inhibiting JAK2/STAT3 signaling pathways.

### 3.1 Pretreatment of ISO improved cellular viability of high-glucose incubated HK-2 cell

**Figure 1.**
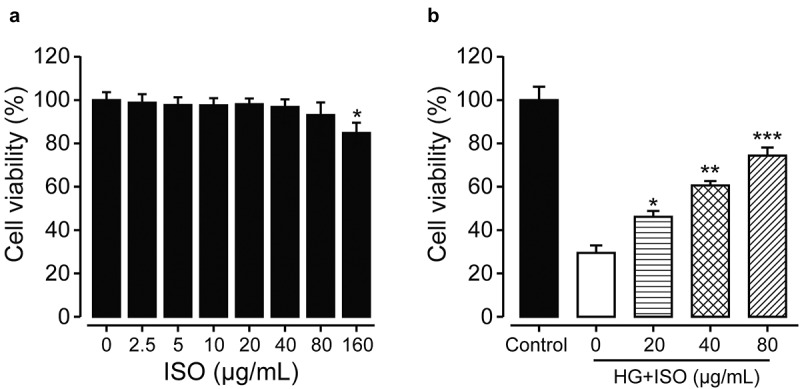
**The protective effects of ISO on cellular viabilities of human renal HK-2 cells** (a) **without or** (b) **with treatment of high glucose**. *p < 0.05, **p < 0.01, ***p < 0.001 compared with saline-treated HK-2 cells without high glucose in Figure 1(a) and with high glucose in Figure 1(b), respectively. All data were presented as mean ± SD (n = 5)

Before we assessed the protective roles of ISO on renal cells, the CCK-8 assay to confirm whether the incubation of different concentrations of ISO will affect cell viabilities of HK-2 cells was firstly conducted. As the data shown in Figure 1, ISO did not obviously decrease the cellular viability of HK-2 within 80 μg/mL compared to the untreated cell group, demonstrating the safe incubated concentration of ISO without significant toxicity to HK-2 cells was between 0 and 80 μg/mL. In addition, overnight high-glucose (30 nmol/L) incubation with HK-2 cells successfully induced cellular injury which further caused a significant decrease of cellular viability (from 100% to 35%). Significantly, the obviously decreased cellular viabilities of the high-glucose incubated HK-2 cells were all reversed under the pre-treatment of ISO at the concentrations of 20, 40, and 80 μg/mL (Figure 1(b)). Moreover, the protective effects of ISO on renal cells were exhibited in a dose-dependent manner. There fore, the pretreatment of ISO could effectively ameliorate the high glucose-induced HK-2 cell damages.

### 3.2 ISO improved oxidative stress in HK-2 cells by increasing oxidation resistance and suppressing the production of ROS

**Figure 2.**
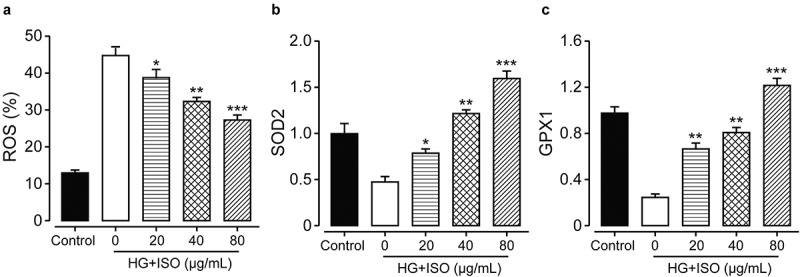
**Effects of treatment of ISO at different concentrations on the antioxidant ability of the HK-2 cell**. The content of (a) ROS and (b) SOD2 and (c) GPX1 in HK-2 cell with the oxidative injury induced by high glucose. *p < 0.05, **p < 0.01, ***p < 0.001 compared with high glucose only treated HK-2 cells. All data were presented as mean ± SD (n = 5)

In order to confirm whether the incubation of ISO could improve oxidative injuries in the human renal cells induced by high glucose, the content of ROS was measured by detecting the intensity of fluorescence using the flow cytometer. As shown in Figure 2(a), the intracellular ROS contents in HK-2 cells were significantly up-regulated under the overnight incubation of 30 mM glucose, and then notably reversed by the incubation of ISO at the final concentration of 20, 40, and 80 μg/mL (all *p < *0.05). Moreover, the expression levels of both GPX1 and SOD2, were further measured to assess antioxidant mechanism of ISO on renal HK-2 cell via Western blotting method. As shown in Figure 2(b-d), the expression levels of GPX1 and SOD2 were both significantly downregulated under overnight incubation of high glucose compared to the HK-2 cells in the normal control group. Significantly, the pre-treatment of all three concentrations of ISO in the renal HK-2 cells all notably reversed the decrease of SOD2 and GPX1, demonstrating that the ISO could efficiently improve the antioxidant abilities of HK-2 cells.

Moreover, we also detected the intracellular GSH content, and results showed that the treatment of high glucose obviously downregulated the GSH in the HK-2 cell which was notably lower than the HK-2 cells in the normal control group (Table 1). Significantly, all three concentrations of ISO increased the GSH content in the HK-2 cell to 75.14 ± 9.12 nmol/mg, 82.54 ± 11.11 nmol/mg, and 112.53 ± 12.81 nmol/mg, respectively, compared with the cells in the negative control (all *p < *0.05). Current data collectively proved that the pre-incubation of ISO could effectively inhibited the production of ROS and improve the oxidation resistance of the human renal HK-2 cell against the high glucose.

**Table 1.** Effects of treatment of ISO at different concentrations on the content of GSH in the high glucose treated renal HK-2 cells

Group	ISO (μg/mL)	GSH (nmol/mg)	DCF Fluorescence intensity (%)
Normal cells	0	131.23 ± 11.23	100
High glucose treated HK-2 cells	0	51.45 ± 11.42	145.22 ± 12.35
	10	75.14 ± 9.12*	131.15 ± 21.56
	20	82.54 ± 11.11**	125.49 ± 15.19*
	40	112.53 ± 12.81***	109.12 ± 11.59***

*p < 0.05, **p < 0.01, ***p < 0.001. vs. high-glucose only treated HK-2 cells (n = 5).

### 3.3 Long-term treatment of ISO improved the diabetic characteristics and renal functions of acute DN model rats

**Figure 3.**
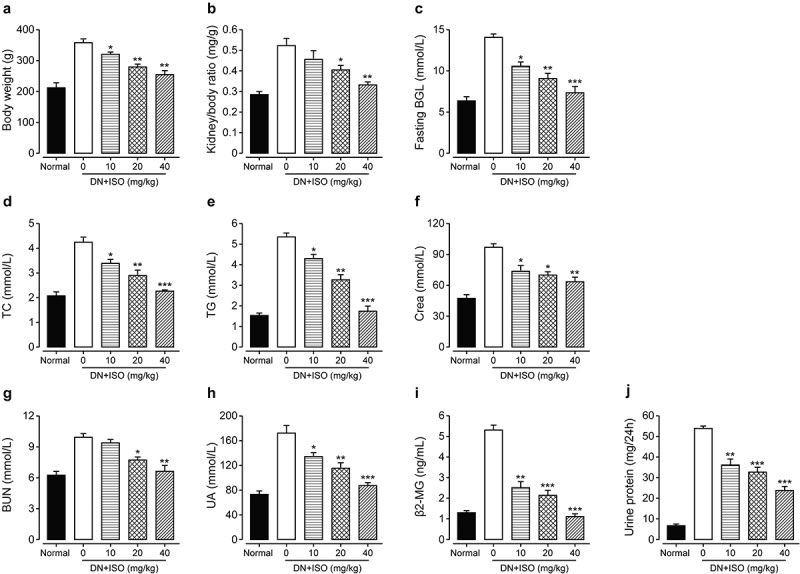
**Effect of long-term administration of ISO at three dosages on the general factors and renal function-related disorders of DN model rats**. The changes of (a) body weight, (b) kidney/body ratio, (c) fasting BGL, and the serum levels of (d) TC, (e) TG, (f) Crea, (g) BUN, (h) UA, (i) β2-MG, and (j) UP/24 h in DN model rats after chronic treatment of ISO. *p < 0.05, **p < 0.01, ***p < 0.001 compared with saline only treated DN model animals. All data were presented as mean ± SD (n = 8)

Initially, DN rats showed obvious systemic reactions included dim fur, hunched posture, and reduced mobility, and exhibited a series of diabetic manifestations such as polydipsia, polyphagia, and polyuria. After long-term administration of ISO at three doses, all clinical signs mentioned above were significantly improved. Furthermore, body/kidney weight ratio, fasting BGLs, TC and TG levels of DN rats were also investigated. As the results shown in Figure 3(a-e), significantly increased body weights, kidney/body weight ratio as well as fasting BGLs were all observed in DN rat compared with the normal healthy ones. Significantly, long-term administration of ISO at all three doses obviously ameliorated these disorders. Moreover, serum levels of TG and TC were also notably improved the ISO treated groups (all *p < *0.05). In addition, the renal functions-related factors, including β2-MG, UA, Crea, BUN, and UP/24 h were all further assessed. As the data shown in Figure 3(f-g), the saline-treated DN model rats showed significantly increased UA, Crea, and β2-MG levels compared to the normal healthy rat. Furthermore, these disorders were all notably improved by chronic administration of ISO at all three dosages (10, 20, and 40 mg/kg). Significantly, the BUN content and urine protein in the DN model rats were also notably upregulated after three doses of ISO treatment (all *p < *0.01). Above data collectively demonstrated that the ISO could effectively ameliorate the disorders of metabolic parameters of DN rat.

### 3.4 Long-term treatment of ISO improved oxidant stress and renal cell apoptosis in acute DN model rat

**Figure 4.**
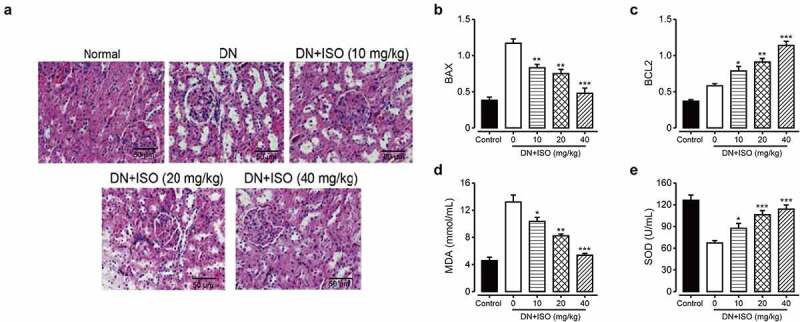
**Effect of long-term administration of ISO at three dosages on the renal tissue changes, oxidative injury-related and apoptosis-related indicators of DN model rat**. (a) H&E-staining images (Scale bar: 50 μm), the expression levels of (b) BCL2 and (d) BAX, and the contents of (D) SOD, (e) MDA and in renal tissues of DN model rat. *p < 0.05, **p < 0.01, ***p < 0.001 compared with saline only treated DN model animals. All data were presented as mean ± SD (n = 8)

In the experiment, oxidative stress induced by high glucose resulted in apoptosis of renal cells. Therefore, we further explored chronic effects of ISO on the apoptosis of renal tissue cells in DN rats. As shown in Figure 4(a), the renal tissue exhibited obviously thickened granular and degeneration of vacuolar, and necrosis on epithelial cell in DN model rat compared with normal healthy ones. Significantly, long-term treatment of ISO showed significant protection on renal tissues compared with saline treated DN rats. Furthermore, we investigated the efficacies of ISO on expression levels of apoptosis-related indicators, including BAX and BCL2, respectively. As shown in Figure 4(d-e), the Bcl2 and BAX expression levels were both significantly downregulated and upregulated, respectively, in DN rats. Significantly, long-term administration of ISO at all dosage all significantly reversed these changes of proteins expression compared to the saline treated DN rats (both *p < *0.05).

We further explore the efficacies of chronic administration of ISO on MDA content, which is an endpoint product of cellular injuries and the formation of free-radical, and also a critical cellular biomarker of the oxidative stress reflecting extent of the oxidative injury. In addition, the SOD, as a key antioxidant enzyme defensing against ROS induced oxidative injury was also measured. As the results shown in Figure 4(b-c), the MDA content and SOD activity in DN model rat were obviously increased and decreased, respectively, compared with those of the normal ones (both *p < *0.01), indicating the upregulated oxidative stress and weakened antioxidant capacity. Significantly, chronic administration of ISO (10, 20, and 40 mg/kg) effectively reversed changes of MDA and SOD levels were all significantly decreased and increased, respectively, which demonstrated that the ISO effectively improved antioxidant ability and down-regulate the oxidative stress in the DN model rat. Above data collectively demonstrated that chronic administration of ISO improved the antioxidant capacity to improve oxidant stress and decrease the renal cell apoptosis to further protect the injured kidney of DN rats.

### 3.5 Long-term administration of ISO downregulated the expressions of fibrosis-related factors of acute DN model rats

**Figure 5.**
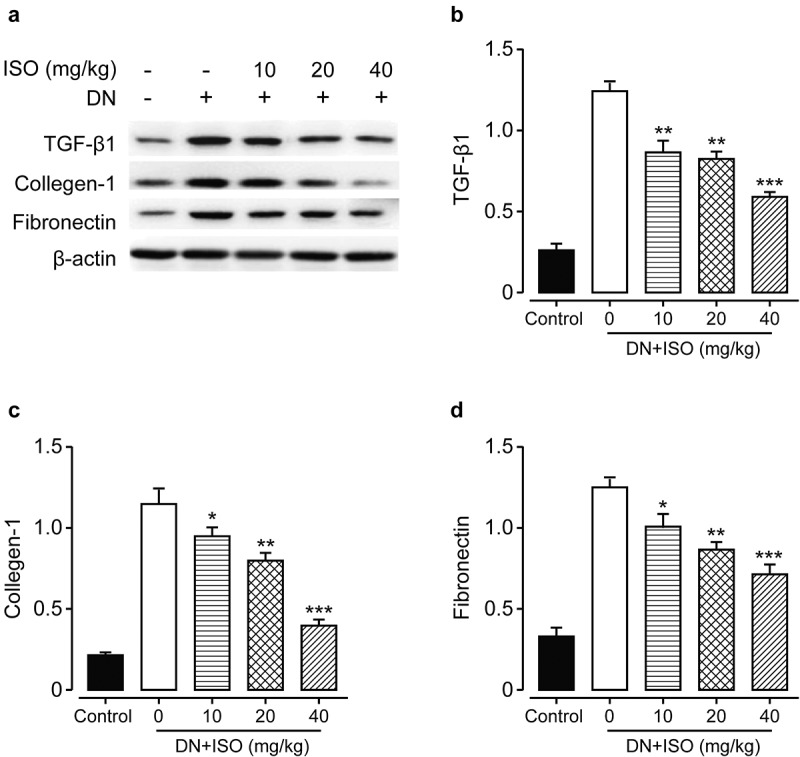
**Effects of 8-week treatment of ISO at three doses on the renal tissue change, the apoptosis and fibrosis-related factors in DKD rats**. (a) The WB analysis of the protein expression levels of (b) TGF-β1, (c) Collagen-1, and (d) Fibronectin protein levels. ***p < 0.001, **p < 0.01 vs. saline-treated DKD model ones. All the above results were showed as Mean ± SD (n = 8)

Renal fibrosis was involved in long-term development of kidney diseases and may ultimately induce the end-stage renal failures. In the current study, we measured the protein expressions of fibrosis-related factors to explore long-term effect of ISO one the fibrosis in DN rats. As shown in Figure 5, the expression levels of fibronectin, Collagen-1, and TGF-β1 were markedly up-regulated in the DN rats treated with saline compared to normal ones (all *p < *0.01). However, these changes were abolished by the treatment of all three doses of ISO (all *p < *0.05). Current results collectively demonstrated that renal fibrosis and injury in DN rat may be ameliorated after consecutive 8-week treatment of ISO via down-regulating the proapoptotic as well as fibrosis-related proteins, and up-regulating the antiapoptotic factor expressions.

### 3.6 ISO protected renal cell inflammation via inhibiting the JAK2/STAT3 signaling pathways of acute DN model rats

**Figure 6.**
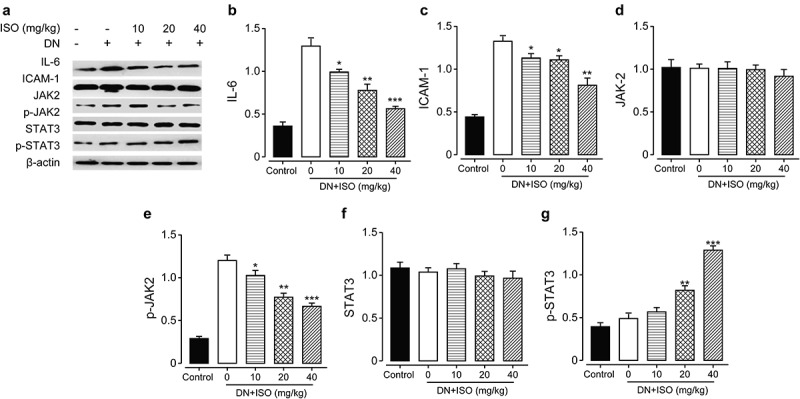
**Chronic effects of ISO treatment on the protein expression levels of JAK/STAT signaling pathway-related indicators in renal tissues of acute DN model rats**. (a) Western blotting gel of the protein expression levels and analysis of (b) IL-6, (c) ICAM-1, (d) JAK2, (e) p-JAK2, (f) STAT-3, and (g) p-STAT-3. *p < 0.05, **p < 0.01, ***p < 0.001 vs. saline-treated DN model rats. All the results were shown as mean ± SD (n = 8)

In present research, the Western blotting method was applied to evaluate the protein expression levels of inflammatory factors, including ICAM-1 and TCH, and JAK2/STAT3 signaling pathways-related indicators in renal tissues. As the results shown in Figure 6, the IL-6 and ICAM-1 levels were both markedly increased in DN model groups compared to the normal healthy rat group (both *p < *0.05). Significantly, upregulated expression of inflammatory factors were reversed by long-term administration of ISO at 10, 20, and 40 mg/kg, and the difference was statistically significant (all *p < *0.05). Considering the relationship between increased inflammatory factors and the activation of JAK2/STAT3 signaling pathways, the expressions of JAK/STAT3 signaling pathways related indicators were further explored. Compared with normal healthy rats, the Western blotting analysis exhibited that relative expressions of p-JAK2 and p-STAT3 in renal tissues of DN ones were obviously upregulated (both *p < *0.05), while expressions of JAK2 or STAT3 did not show significant change. Significantly, these upregulated factors were markedly decreased by chronic treatment of ISO at all three dosages. Above data collectively suggested the protection effect of ISO on renal tissues of DN rats might rely on downregulating JAK2/STAT3 signaling pathways to inhibit the protein expression levels of down-stream inflammatory indicators.

## 4. Discussion

Diabetes mellitus (DM) is a common chronic lifelong disease and is clinically characterized by the coexistence of hyperglycemia and multiple chronic complications with a high genetic predisposition that endangers human health [26,27]. As a metabolic disease, the pathogenesis of DM involves multiple factors, such as abnormal genetics and excessive cytokines, and is ultimately characterized by abnormal glucose metabolism due to absolute or relative deficiency and dysfunction of insulin secretion [27,28]. Diabetic patients under long-term chronic hyperglycemia state suffer the damage even the failure of kidney, eyes and heart, and other organs [29,30]. Chronic vascular complications of DM are mainly divided into microvascular and macrovascular complications, mainly including diabetic retinopathy (DR) in 31.5% and diabetic kidney disease (DKD) in 39.7% [31]. DKD is one of the common and critical chronic vascular complications in the terminal stage of DM disease [32]. Patients with DM exhibit abnormal glucose and lipid metabolism, and glomerular cell proliferation, which immediately leads to abnormal glomerular hypertrophy, basement membrane thickening, glomerular sclerosis, and other pathological changes, and ultimately leads to DKD [11,33]. Moreover, DKD is clinically characterized by progressive urinary protein increase, edema, low protein, glucose and lipid metabolism disorders, irreversible decline in renal function, and the incidence and mortality of cardiovascular disease [17,34]. The pathogenesis of DKD is very complex, and the exact pathogenesis remains undefined [4]. In recent years, clinical and experimental studies have shown that multiple factors such as genetics, abnormal glucose metabolism, hemodynamic changes, and inflammatory mediators may play important roles in the pathogenesis of DKD, and each factor does not exist in isolation and can interact with each other to lead to renal structural abnormalities and the occurrence and development of DKD [1,2,35]. Due to the intricate pathogenesis of DKD and mutual influence, although active lowering of blood glucose can slow down disease development, the overall therapeutic effect is still not satisfactory.

*Glycyrrhiza uralensis* is a perennial herb legume, and its dried roots and rhizomes contain a variety of flavonoid components, which are widely used in the treatment of sore throat, painful sores, and gastrointestinal ulcers [36,37]. Isoliquiritigenin (ISO) is a flavonoid extracted from the roots of *Glycyrrhiza uralensis*, which belongs to the hydroxyl chalcone class of compounds [21]. Studies have found that ISO has good anti-tumor, antioxidant, and other pharmacological activities, and has protective effects on the heart and brain [24,25]. Due to the wide range of anti-inflammatory activity, clear mechanism, and enhanced activity of modifiers, ISO has the potential to be developed as a new drug for treating DKD.

In the present study, we first investigated the protective effects of ISO at different final concentrations on normal human renal cells under the stimulation of high glucose. As results shown in Figure 1, HK-2 cells exhibited well drug tolerance when the final concentration of ISO was below 80 μg/mL, and of which the cell viability also did not showed significant decrease compared with the untreated normal HK-2 cells group. Further overnight incubation of high-glucose (30 nmol/L) with HK-2 cells induced obvious cellular injuries which resulted in an obvious decrease of cellular viability. Moreover, the cellular viability of high-glucose incubated HK-2 cells was reversed by pretreating the ISO at final concentrations of 20, 40, and 80 μg/mL (Figure 1). Hence, above data collectively suggested that pre-treatment of ISO could effectively ameliorate the high glucose-induced damages and increase the viabilities of HK-2 cells.

Oxidative stress refers to the process in which the body suffers from various harmful stimuli, such as long-term high glucose state, metabolic disorders, or excessive generation of highly reactive molecular substances in the body, such as ROS, and the degree of oxidation far exceeds the scavenging ability of endogenous antioxidant defense systems to oxides, disrupting the dynamic balance state of oxidative system, and antioxidant system, resulting in cell and tissue damage [38,39]. Under normal physiological conditions, antioxidant systems in the body such as SOD2, GSH, and ascorbic acid are able to scavenge ROS produced by oxidation-32 stress to maintain the homeostasis of oxidation-reduction reactions in the body. Many complications of DM are associated with oxidative stress, such as DN and DR. Excessive accumulation of ROS in the body can directly damage podocytes, resulting in the loss of a large amount of negative charges from the basement membrane and disrupting the integrity of the glomerular filtration membrane barrier structure, resulting in protein leakage. Chronic hyperglycemia can directly stimulate the production of ROS. Excessive ROS promote the initiation or waterfall amplification of oxidative stress in renal tissue. Excessive generation of AGEs, AR activation, and other changes can lead to the production of a large number of oxygen free radicals. At the same time, high glucose can also lead to glycosylation of antioxidant enzymes, with decreased activity and expression and decreased scavenging capacity. In combination with a variety of pathways, it is involved in renal tissue damage, such as affecting target tissue metabolism, matrix remodeling, hemodynamic changes, inflammatory response, podocyte injury, and interstitial fibrosis, and finally develops into end-stage renal disease. It has been confirmed that antioxidant therapy is one of the effective means of treating DN. In addition, ROS can also destroy the glycoprotein of endothelial cells and further aggravate endothelial cell injury.

To confirm whether the pre-incubation of ISO at different final concentrations could effectively improve oxidative injuries in HK-2 cells induced by high glucose, we measured the ROS contents and the results showed that intracellular ROS content in HK-2 cell was obviously upregulated below 30 mM glucose incubation. The overly increased ROS content was notably reversed by ISO incubation at all three final concentrations in a clear dose dependence (20, 40, and 80 μg/mL, all *p < *0.05). Moreover, the changes of the protein expression level of SOD2 and GPX1 in ISO groups could demonstrate that incubation of ISO could upregulated the contents of GSH, GPX1, and SOD2 to increase the clearance of ROS, and then improve the cell viability of HK-2 cells against the high-glucose. Similar results were also shown in the DN model rats *in vivo*, ISO effectively improved antioxidant abilities, and down-regulate oxidative stress in the DN rat.

Chronic experiment was further performed to assess the effects of long-term treatment of ISO on the diabetic characteristics as well as renal functions of acute DN model rats. Significantly, three dosages of ISO all effectively improved disorders, including body/kidney weight ratio, fasting BGLs, TG and TC levels of DN ones. Moreover, the renal functional indicators, including β2-MG, UA, Crea, and BUN, were all obviously improved by chronic administration of ISO at all three dosages (10, 20, and 40 mg/kg). Hence, the above results collectively demonstrated that the ISO could effectively ameliorate the disorders of metabolic parameters of acute DN rats.

Under the influence of long-term high glucose, the morphological structure of the kidney will also be significantly changed. The results of H&E staining in this study showed that the glomeruli of rats became larger, proliferated, the renal tubules were turbid and extensively vacuolated, inflammatory cells infiltrated, and the tissues and cells were disorganized under the light microscope, indicating that the model rats held obvious pathological damages and developed renal fibrosis. After the treatment with different doses of ISO, H&E staining showed that glomerular matrix proliferation was alleviated at different levels, inflammatory cell infiltration was gradually reduced, and fibers were smaller than those in the model group, indicating that ISO could alleviate the pathological changes of the kidney to a certain extent and improve renal fibrosis. Unsurprisingly, the expressions of fibrosis-related proteins, including TGF-β1, fibronectin and Collagen-1, were markedly decreased in all three ISO groups compared to saline-treated DN ones (all *p < *0.01). However, the above changes were markedly abolished via the treatment of ISO (all *p < *0.05). Moreover, combined with data of expression levels of apoptosis-related factors, we collectively demonstrated renal injury and fibrosis in acute DN rats could be ameliorated after 8-week treatment of ISO via inhibiting expressions of proapoptotic and fibrosis-related proteins, and increasing antiapoptotic factor expression to further protect the impaired kidney of DN model rats.

Recently, numerous studies have implicated the JAK2/STAT3 signaling pathway in the progression of DN and various diabetic characters such as hyperglycemia, angiotensin II, and AGEs can also activate the JAK/STAT pathway. Moreover, many evidences suggested that the JAK2/STAT3 pathway could act on glomerular mesangial cells. Therefore, the activated JAK/STAT pathway may further stimulate the excessive proliferation and growth of GMCs, resulting in diabetic kidney injury. In this study, we analyzed that the IL-6 and ICAM-1, as inflammatory cytokines and downstream of JAK2/STAT3 signaling pathway, were markedly down-regulated compared to those of the saline-treated ones. Further, WB data exhibited that the expressions of p-STAT3 and p-JAK2 in renal tissues of DN ones were significantly upregulated (*p < *0.05) while in three ISO groups were all obviously reversed. The above results collectively demonstrated that ISO could down-regulate the signaling pathway of JAK2/STAT3 to inhibit the protein expressions of downstream inflammatory factors to protect the kidney of acute DN model rats.

## 5. Conclusion

In conclusion, long-term administration of ISO at all three dosages (10, 20, and 40 mg/kg) effectively improved STZ-induced acute renal injuries in DN model rats via improving renal fibrosis, oxidative stress, and inhibiting JAK2/STAT3 signaling pathway. The results of our study also provided adequate mechanisms for the subsequent clinical applications of ISO.

## Data Availability

All data generated or analyzed during this study are included in this article.

## Highlight

- Isoliquiritigenin improved cellular viability of high-glucose incubated HK-2 cell.
- Chronic Isoliquiritigenin treatment ameliorated renal functions of diabetic rats.
- Isoliquiritigenin attenuated oxidant stress and renal cell apoptosis of diabetic rats.
- Isoliquiritigenin down-regulated the expressions of fibrosis-related factors.
- Isoliquiritigenin suppressed inflammation via inhibiting the JAK2/STAT3 pathway.
